# Pediatric Reconstruction of Full-Thickness Dog Bite Scalp Avulsion with a Combination of Acellular and Matrix Products: A Case Report

**DOI:** 10.3390/medicina60111838

**Published:** 2024-11-08

**Authors:** Francesca Grussu, Guido Ciprandi, Federico Lo Torto, Diego Ribuffo, Mario Zama

**Affiliations:** 1 Plastic and Maxillofacial Surgery Unit—Bambino Gesù Children Hospital—Rome, 00120 Vatican City, Vatican City State; francesca.grussu@opbg.net (F.G.);; 2 Department of Plastic Reconstructive and Aesthetic Surgery, Policlinico Umberto I, Sapienza University of Rome, 00161 Rome, Italy

**Keywords:** scalp, dog bite, pediatric

## Abstract

The reconstruction of large full-thickness scalp injuries represents a great challenge in pediatric plastic surgery. Epidermal–dermal substitutes come to the rescue when traditional surgical strategies are not suitable. Recently, the new Integra MicroMatrix UBM particulate has arisen on the market. This is an extracellular matrix derived from the porcine urinary bladder matrix (UBM) in which the particulate structure provides close contact to the wound bed. We present the concomitant application of Integra DRT and MicroMatrix for the reconstruction of a full-thickness subtotal scalp defect in a child mauled by a dog.

## 1. Introduction

The reconstruction of large full-thickness scalp injuries represents a great challenge in pediatric plastic surgery [1,2,3,4,5,6]. Skin grafting, local flaps, tissue expansion, and free tissue transfers are all good options, although not always suitable. In these cases, epidermal–dermal substitutes come to the rescue, replacing or improving the functions of human skin and regenerating damaged tissue [7,8,9,10,11,12,13,14]. The Integra dermal regeneration template has been shown to be an essential step of the scalp reconstructive ladder. It is used for the reconstruction of traumatic wounds, burn injuries, and post-oncological resections [15,16]. Recently, the new Integra MicroMatrix UBM particulate has arisen on the market. This is an extracellular matrix derived from the porcine urinary bladder matrix (UBM) in which the particulate structure provides close contact to the wound bed. The purpose of this case report is to present the complementary application of Integra DRT and MicroMatrix for the reconstruction of a full-thickness subtotal scalp defect in a child mauled by a dog.

## 2. Clinical Case Report

After the initial emergency admission to an adult hospital, where was done a first tentative to cover the scalp with a skin-substitute, a 2 years-old female was referred to our Plastic and Maxillofacial Surgery Unit at Bambino Gesù Children’s Hospital in Rome.where it was determined that her scalp should be covered with a skin substitute, a two-year-old female was referred to our Plastic and Maxillofacial Surgery Unit at Bambino Gesù Children’s Hospital in Rome. The girl presented an evident near-total avulsion of her scalp after a family dog attack, with only the occipital region preserved. A careful examination also revealed the laceration of the upper third of her right ear, with an avulsion of the entire helix region and part of the concha, together with the complete removal of the parietotemporal scalp.

The little girl was sleeping on the sofa when the animal attacked her, biting her scalp, so there was no defensive action and only her grandmother rescuing her prevented a more serious outcome.

**Figure 1:**
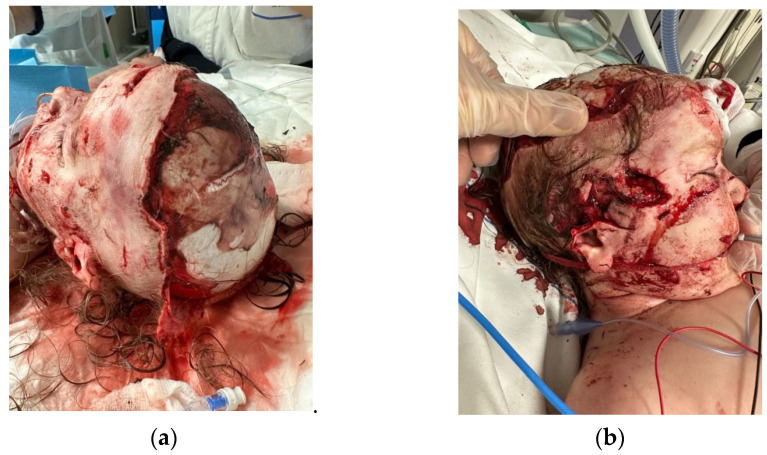
A two-year-old female referred to our Plastic and Maxillofacial Surgery Unit at Bambino Gesù Children’s Hospital in Rome presented the evident near-total avulsion of her scalp after a family dog attack (**a**) and the laceration of the upper third of her right ear, with an avulsion of the entire helix region and part of the concha, together with the complete removal of the parietotemporal scalp (**b**).

Local flaps were not achievable due to the 48 h delay from the dog bite and the consequent increasing probability of local contamination/infection, the very large size of the defect, and the traumatic temporal artery damage. Likewise, tissue expansion was not an immediate option given the lack of available tissue. We considered performing a free flap, but we elected to try an easier option, leaving the free flap as a rescue option (Figure 1a,b).

**Figure 2:**
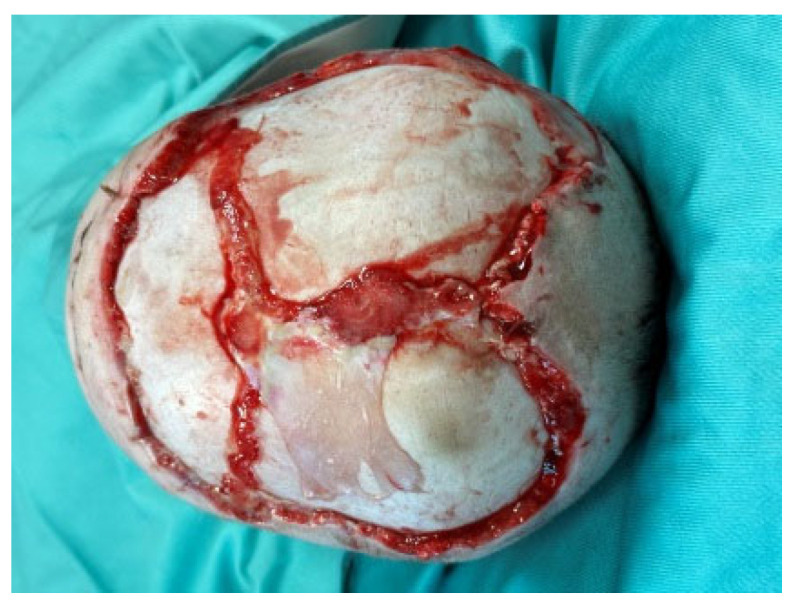
After applying an intense wound hygiene protocol, all five layers of the scalp were absent and only a small portion of the periosteum was present.

After applying an intense wound hygiene protocol, the i.o. observation of the decontaminated and cleansed wound bed allowed us to observe that all five layers of the scalp were absent and only a small portion of the periosteum was present. The mesenchymal tissue regeneration was disordered and at times hyperplastic; this is commonly reported as a consequence of local critical bacterial contamination (Figure 2) [17].

**Figure 3:**
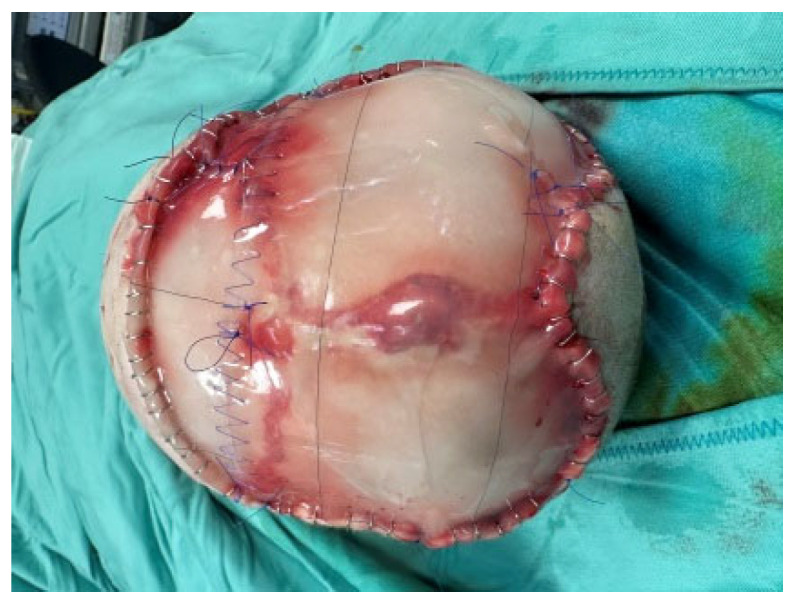
After debridement, we applied two re-shaped Integra Dermal Regeneration Template double layer sheets directly onto the bone.

**Figure 4:**
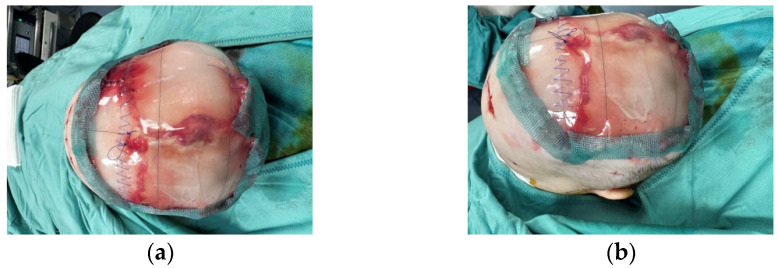
Due to contamination and dryness, we applied a hydrophobic dressing with broad-spectrum irreversible bacterial binding action. Upper (**a**) and lateral (**b**) visions of the dressing.

Thus, the scalp defect was debrided, as were the skin circumferential margins, and we applied two re-shaped Integra Dermal Regeneration Template double layer sheets directly onto the bone without burring-out the outer bony cortex (Figure 3). We classified this complex wound as contaminated and partially dirty. That is why we chose to protect the entire suture line with Cutimed Sorbact gel, a hydrophobic technology with broad-spectrum irreversible bacterial binding action (Figure 4a,b) [18].

**Figure 5:**
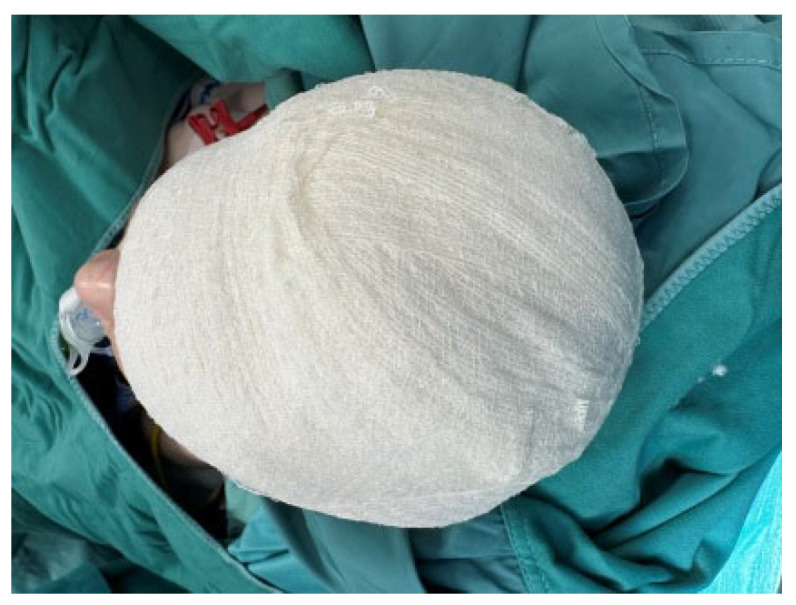
We covered the scalp with an easily removable polyurethane foam fixed with Mollelast haft latex-free tape to protect it from external contamination.

Small fenestrations were made to prevent fluid and blood postoperative buildup, and we covered the scalp with an easily removable polyurethane foam to allow excess fluids to pass away from the wound and to protect it from external contamination, preventing the risk of an SSI. The casket was completed and fixed with Mollelast haft latex-free tape (Figure 5).

The dressing (Mepilex Lite foam, Molnlycke) was changed every 3 to 5 days for 21 days. After 21 days, in order to obtain better coverage of the bone, we applied another sheet of Integra double layer sheets with the same postoperative care mentioned above.

**Figure 6:**
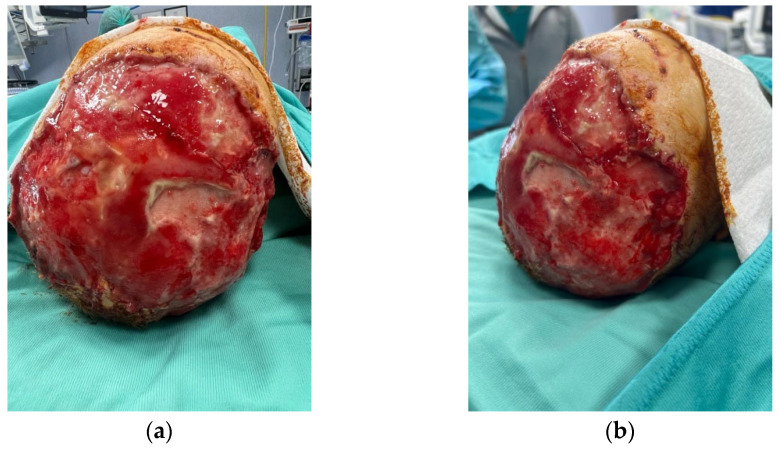
After the second step, we reported the satisfying growth of the underlying neo-dermis, but the surface still presented some irregularity. Upper (**a**) and lateral (**b**) visions.

After this second step, we reported the satisfying growth of the underlying neo-dermis and good adherence to the residual skin limits, but the surface still presented some irregularities and deep areas unsuitable to guarantee a final good skin engraftment (Figure 6a,b).

**Figure 7:**
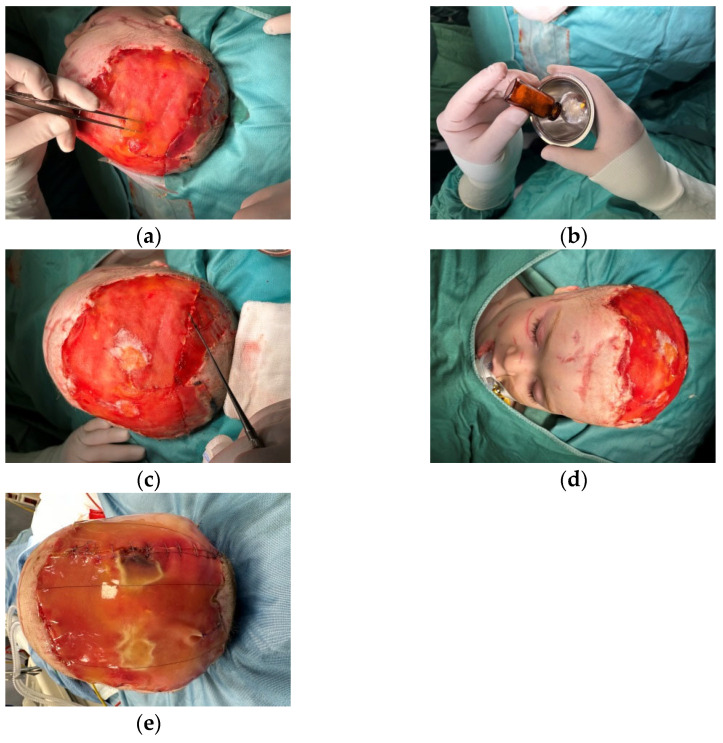
We treated the tissue losses, (**a**) filling the gaps with the Integra MicroMatrix UBM standard particulate ACell (**b**–**d**), covered with a third sheet of Integra double layer sheets (**e**).

We decided to treat these tissue losses by filling the gaps with the Integra MicroMatrix UBM standard particulate ACell, covered with a third sheet of Integra double layer sheets (Figure 7a–e).

MicroMatrix was produced from UMB (urinary bladder) technology and consists of an extracellular scaffold (EMC) that supports the shift in the host immune response from a scar-like response to a remodeling that facilitates the deposition of vascularized tissue. This kind of technology has been proven to support a prompt anti-inflammatory response, completely reabsorb the matrix that is incorporated, mobilize host cells, and promote the deposition of new tissue through constructive local remodeling. In the recent past, this M-UBM has been successfully used in diabetic feet, chronic pressure ulcers, venous leg ulcers, irregular wound beds, and in undermined wound’s borders.

**Figure 8:**
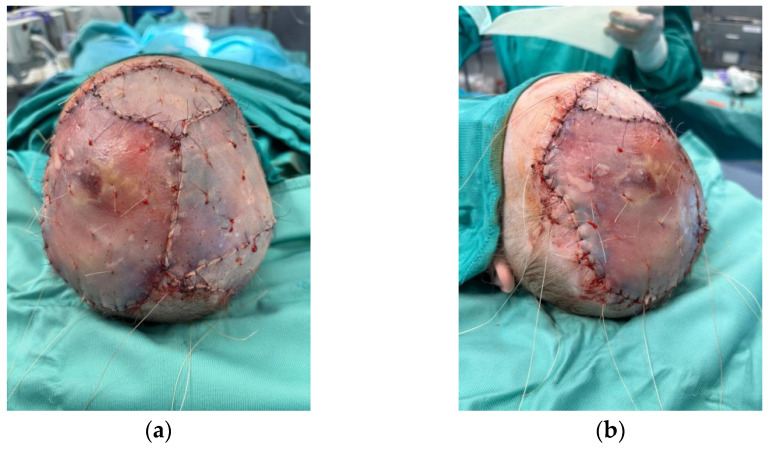
The split-thickness skin grafts after obtaining a smooth and matured surface after 3 weeks. Upper (**a**) and lateral (**b**) visions of the result.

After 3 weeks, we obtained a smooth and matured surface, which was grafted with a split-thickness skin graft (Figure 8a,b).

At this stage and twice a day for 15 consecutive days, all the sutures were protected and activated with Vulnamin spray, a composition consisting of sodium hyaluronate, and six selected amino acids for collagen production. All the dressings were performed at home by the mother, with the help of intense psychological assistance.

**Figure 9:**
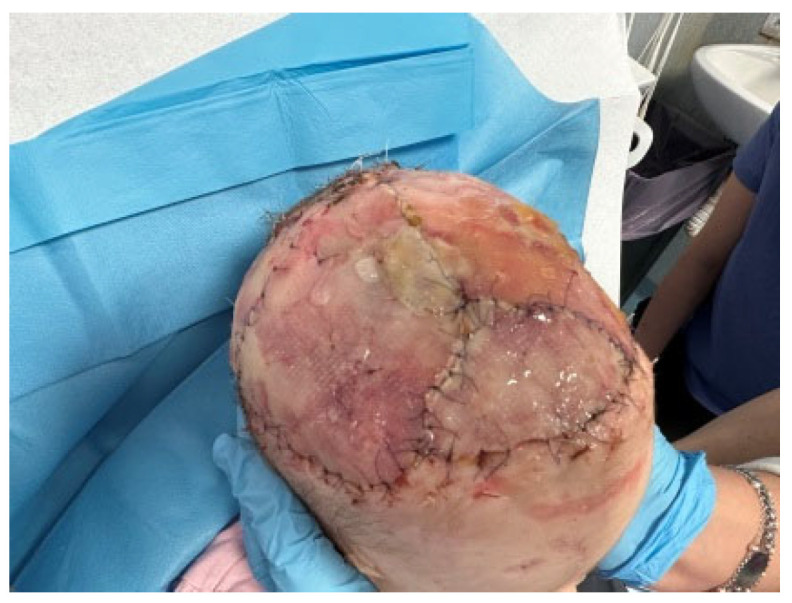
Results after 7 days with an adequate take of the skin graft.

**Figure 10:**
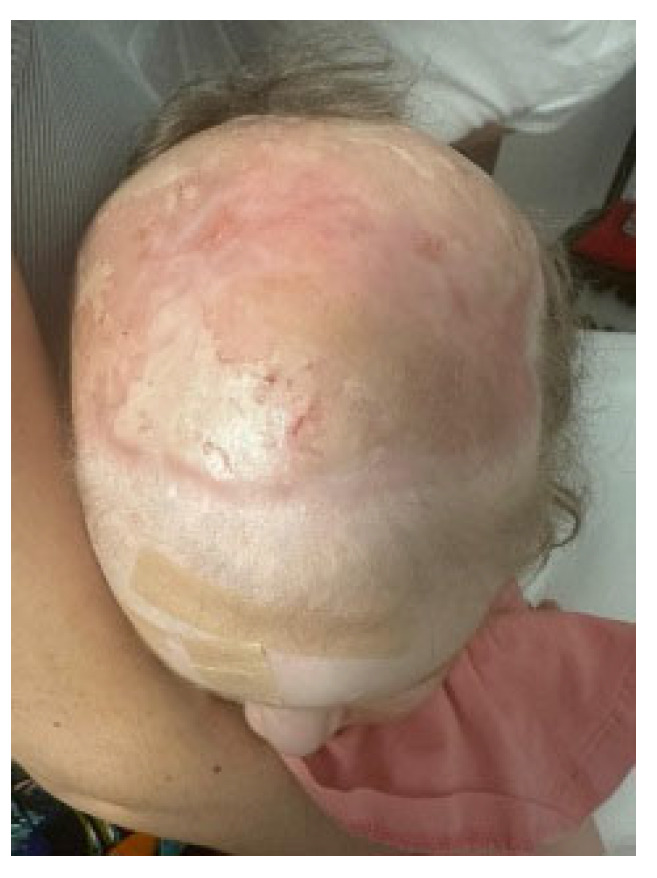
6 months after the original injury, the patient demonstrated pliable, uniform, and stable coverage.

Adequate intake of the skin graft was observed 7 days later (Figure 9). 6 months after the original injury, the patient demonstrated pliable, uniform, and stable coverage. It is certain that the patient will undergo further reconstructive procedures due to permanent post-traumatic alopecia (Figure 10).

## 3. Discussion

This is the first pediatric reported case of a scalp avulsion staged reconstruction fully healed by using paired skin substitutes of a different origin. The reconstruction of the scalp in children is a very challenging procedure due to tissue deficiency and poor elasticity. The immediate coverage of the calvaria was mandatory in order to avoid dehydration and the development of severe deep soft tissue infection and consequent skull osteomyelitis [1,2,3,4,5,6].

For this reason, tissue expansion, local flaps, and free tissue transfers were the first and most effective options. However, they are not always suitable, such as in the case described.

The use of a dermal matrix followed by skin grafting has been described in cases of scalp reconstruction in which other options were not available [7,8,9,10,11,12,13,14].

The Integra dermal matrix is nowadays properly used for the reconstruction of scalp defects with satisfactory results [15,16]. Regardless, the irregularity of the wound bed was one of the more frequent drawbacks experienced in the reconstruction of the traumatic wound of the scalp, even after the use of the Integra dermal matrix. The irregularity of the wound bed could compromise the stability and the engraftment rate of the skin graft.

Hence, we present our result using Integra, particularly the new product Integra MicroMatrix UBM particulate, which is a particulate compound of the extracellular matrix derived from the porcine urinary bladder matrix. Due to its structure, it provides intimate contact with the wound bed and more flexibility, making it ideal for the treatment of irregular surfaces. As described in our case, we used a combination of the Integra double layer sheets, put on the wound bed in three surgical steps, and the Integra MicroMatrix in the last step for the coverage of the remaining irregular area of the wound bed. We applied Integra directly onto the bone without burring-out the outer bony cortex, as suggested by Komorowska-Timek et al. [19]. Although there is feasibility in this technique for promoting the development of adequate granulation tissue for grafting, we preferred not to apply it because, in this case, it would have increased the risk of surface irregularity. Moreover, in the pediatric age, sutures have important regranulation activity that immediately supports the dermo-epidermal substitute and amplifies its scaffold activity by spreading over the entire surface.

We obtained smooth, well-vascularized and stable underlying tissue, as evidenced by the take of the split-thickness skin graft, which remained stable in the long-term follow-up of six months.

## 4. Conclusions

In our experience, Integra DRT and Integra MicroMatrix can be used together for the management of a full-thickness complex wounds with irregular tracts, funds, and fistulas, providing coverage through close contact with all the areas of the restored wound.

A complete pediatric wound care program starting from wound hygiene up to the accurate protection of skin grafts is essential for final success without complications.

The combination of acellular and matrix products represents a valid option for the treatment of complex scalp-contaminated dirty wounds even in early life, and it can be added as an integral part of the reconstructive ladder in pediatric reconstructive surgery.
